# Intelligent Screening of Prostate Cancer Individuals Using an Enzyme‐Assisted Multicolor Visualization Platform

**DOI:** 10.1002/advs.202408825

**Published:** 2024-11-08

**Authors:** Ruomei Teng, Ming Li, Zikang Chen, Jianli Lin, Yuhan Zhang, Hang Li, Zejun Yan, Dingyuan Zhang, Caiping Ding, Youju Huang

**Affiliations:** ^1^ College of Material Chemistry and Chemical Engineering Key Laboratory of Organosilicon Chemistry and Material Technology Ministry of Education Department of Orthopedics Hangzhou Normal University Affiliated Hospital Hangzhou Normal University Hangzhou Zhejiang 311121 China; ^2^ Department of Urology & Nephrology The First Affiliated Hospital of Ningbo University 59, Liuting Street Ningbo Zhejiang 315010 China

**Keywords:** intelligent diagnosis, multicolor visualization, prostate cancer, RGB colors, urine sample

## Abstract

Rapid and intelligent identification of prostate cancer (PCa) is critical for early diagnosis. Herein, a convenient, reliable, and intelligent strategy is proposed to screen PCa individuals through indirectly quantifying sarcosine (Sar), an early indicator of PCa, in clinical urine samples. Success is achieved by integrating sarcosine oxidase (SOX) as a specific recognition unit; nanozyme‐assisted multicolor intelligent visualization platform as a signal reporter. With the Fe‐MOFs and peroxidase, the synergetic action of SOX and response gold nanorods (Au NRs) is controlled etched to exhibit a multicolored signal. The sensor exhibits excellent linearity with Sar within 1–60 × 10^−6^ m, boasting a remarkable detection limit of 0.12 × 10^−6^ m. The RGB value of the display color can be directly extracted using a mobile phone camera. PCa diagnosis can be swiftly made (within 15 min) and directly by identifying two RGB colors (*R* < 175 or *B* > 135). The enzyme‐assisted multicolor intelligent visualization platform is adept at detecting minute differences in Sar concentration in urine samples between PCa patients and healthy individuals. The concept of enzyme‐assisted multicolor sensing can be further expanded by modifying the type of immobilized enzymes, providing a valuable guideline for the rational design of multiple probes to measure specific biomarkers in biological samples.

## Graphical abstract

Rapid and intelligent prostate cancer (PCa) identification is crucial for early diagnosis. A strategy is proposed using sarcosine quantification in urine, achieving intelligent multicolor visualization platform. The RGB value can be extracted via mobile phone camera, enabling swift PCa diagnosis. This platform detects minute sarcosine concentration differences and can be expanded for measuring specific biomarkers in biological samples.

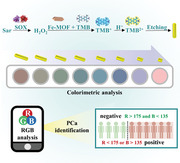

## 1 Introduction

Prostate cancer (PCa) is one of the most prevalent types of cancer that significantly impacts men's health.^[^ ^1^ ^]^ In its early stages, PCa often exhibits no apparent symptoms, making early detection vital for enhancing life quality and reducing mortality rates.^[^ ^2^ ^]^ Prostate‐specific antigen (PSA) stands as the most frequently utilized biomarker for prostate cancer (PCa).^[^ ^3^, ^4^ ^]^ Nevertheless, PSA screening often encounters a challenging gray zone ranging from 4 to 10 ng mL^−1^, making it difficult to precisely diagnose early‐stage prostate cancer within this range. The presence of sarcosine (Sar) in the urine serves as a simple and noninvasive tool for the early diagnosis of PCa.^[^ ^5^, ^6^ ^]^ Generally, sarcosine levels in human urine range from 1–3 × 10^−6^ m under normal physiological conditions, with only a significant increase observed during the pathological process of PCa.^[^ ^7^ ^]^ As an alternative to blood analysis, urine analysis has gained popularity as a straight forward and minimally invasive screening procedure in modern medicine. This type of information offers clinicians valuable insight into a patient's overall health status. Furthermore, advancements such as point‐of‐care testing devices and “smart toilets” have made urine analysis more convenient for patients, facilitating self‐diagnosis.^[^ ^8^, ^9^, ^10^ ^]^ However, urine analysis for PCa detection presents limitations and challenges. One constraint is the potential for false positives or false negatives, as the presence of Sar in the urine can also be influenced by factors unrelated to PCa. Additionally, urine analysis accuracy may vary depending on the sensitivity and specificity of the testing method used. Despite the convenience of point‐of‐care testing devices and smart toilets, there is still a need for proper validation and standardization of these technologies to ensure reliable results. It is therefore essential to detect Sar content in urine rapidly and intelligently to diagnose and treat PCa at an early stage.

The most commonly employed techniques for Sar determination encompass gas chromatography (GC),^[^ ^11^ ^]^ liquid chromatography coupled to mass spectrometry (LC/MS),^[^ ^12^ ^]^ high‐performance liquid chromatography coupled to mass spectrometry (HPLC/MS),^[^ ^13^ ^]^ capillary electrophoresis (CE).^[^ ^14^ ^]^ While colorimetric methods boast advantages such as intuitiveness, rapidity, simplicity, and repeatability, they still lack the sophistication and sensitivity of techniques like GC, LC, and MS. These more complex methods offer higher resolution and accuracy in Sar determination, but they also present drawbacks, including expensive instrumentation, extensive sample pre‐treatment, and lengthy analysis procedures. Therefore, the challenge lies in striking a balance between colorimetric convenience and the precision of other detection techniques. The key to success in designing rapid and intelligent colorimetric methods roots in creating a response model that utilizes an integrated enzyme as a specific recognition unit and couples intuitive visual signals as reporters. For example, many researchers use sarcosine oxidase (SOX) and peroxidase (POD)‐active materials (HRP, Fe_3_O_4_@SiO_2_@NiCo_2_S_4_, SiO_2_@TiO_2_, etc.) to oxidize 3,3′,5,5′ ‐tetramethylbenzidine (TMB) for color biosensor development.^[^ ^15^, ^16^, ^17^ ^]^ Ultimately, the use of these materials helps researchers to develop reliable and efficient color biosensors for Sar analysis.

The above research on nanoenzymes has gained increasing attention due to their remarkable physical and chemical properties, cost‐effectiveness, high stability, and convenient storage.^[^ ^18^, ^19^, ^20^ ^]^ Among the various nanoenzymes, iron‐based nanocatalysts have potential for catalyzing Fenton reactions and facilitating H_2_O_2_ conversion into hydroxyl radical (·OH).^[^ ^21^ ^]^ And metal‐organic skeletons (MOFs) and its derivatives, as a novel type of porous material, possess a clear coordination network, mesoporous structure and adjustable porosity. They can simulate the active center of natural enzymes while providing a hydrophobic environment, which is conducive to the enzymatic reaction.^[^ ^22^, ^23^ ^]^ To enhance the conversion of H_2_O_2_ to ·OH and improve detection sensitivity, a metal‐organic framework with iron (Fe‐MOF) nanozyme, based on the principle of Fenton reaction and peroxidase, emerges as an excellent choice as a peroxidase mimics.^[^ ^24^, ^25^, ^26^ ^]^ This is because the Fe‐MOF nanozyme exhibits catalytic activity comparable to natural peroxidase, shows minimal susceptibility to external influences, and possesses exceptional reaction efficiency.^[^ ^27^ ^]^ As such, the Fe‐MOF nanozyme is a potential candidate for a wide range of applications.

Simultaneously, the oxidized TMB color modification presents a spectrum of blue shades, resulting in limited distinguishability and significant disparities in intuitive differentiation. The human visual system can discern approximately 10 million distinct colors, encompassing hue, brightness, and saturation. However, perceiving changes in brightness and saturation proves more challenging than hue changes.^[^ ^28^ ^]^ Therefore, it is advantageous to enhance the sensitivity and precision of colorimetric detection by converting the difficult‐to‐detect modifications in brightness and saturation caused by the analyte into readily perceptible variations in hue. Our previous research has shown that noble metal nanoparticles, such as gold nanorods (Au NRs) and gold nanopyramids (Au NBPs), exhibit a high extinction coefficient.^[^ ^29^, ^30^, ^31^, ^32^, ^33^ ^]^ Their longitudinal localized surface plasmon resonance (LSPR) is particularly sensitive to aspect ratio changes. The potent oxidizing agent TMB^2+^ by nanozyme can induce alterations in shape and size, consequently affecting their longitudinal LSPR. Characterized by vivid color transformations, these nanoparticles serve as an excellent material for multi‐color visualizations.^[^ ^34^, ^35^ ^]^ By harnessing the vivid color transformations induced by analyte‐induced shape and size alterations, these nanoparticles enable the development of sensitive and precise detection methods for a wide range of substances. Consequently, researchers are continuously exploring ways to improve colorimetric methods, such as designing rapid and intelligent response models, to achieve a balance between convenience and accuracy in Sar determination.

**Scheme 1:**
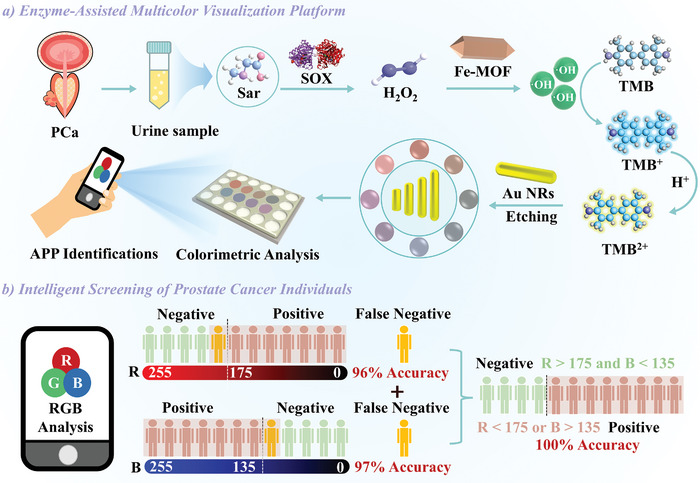
a) Schematic diagram illustrating the enzyme‐assisted multicolor visualization platform involving SOX and Fe‐MOF for etching Au NRs, facilitating a multicolor visualization platform for Sar detection. (b) Schematic diagram demonstrating the integration of two RGB colors for intelligent PCa diagnosis.

Based on these findings, an intelligent screening method for PCa individuals was designed using an enzyme‐assisted multicolor visualization platform (**Scheme** **1**). The specificity of an enzymatic reaction between SOX and Sar avoid interference from structurally similar biomolecules in urine. Meanwhile, Fe‐MOF with peroxidase‐like activity further reacts with H_2_O_2_ to produce reactive oxygen species to oxidize TMB, ultimately transforming it into TMB^2+^. This process results in Au NRs etched into a spectrum of vibrant colors. The color variation could be promptly distinguished by the coupled Au NRs indicators, giving an indirect color response toward Sar. The RGB value of the display color can be easily extracted by taking a photo with a smartphone. When *R* < 175 or *B* > 135, PCa can be diagnosed quickly (within 15 min) in a simple and intuitive manner. Furthermore, the longitudinal LSPR peak shift of Au NRs is linearly correlated with Sar levels, enabling accurate Sar quantification in urine samples. This enzyme‐assisted multicolor visualization intelligent platform was specifically applied to screen PCa patients by quantifying the minute concentration change of Sar in urine samples. This approach provides a theoretical basis for the development of an early rapid diagnosis kit for clinical PCa. Moreover, advancements in smartphone technology and image analysis algorithms could further streamline the detection process and enhance the user experience, making the rapid diagnosis kit more accessible and user‐friendly for both patients and healthcare professionals.

## 2 Results and Discussion

### 2.1 Synthesis and Characterization of Nanoprobes for an Intelligent Multicolor Visualization Platform

**Figure 1:**
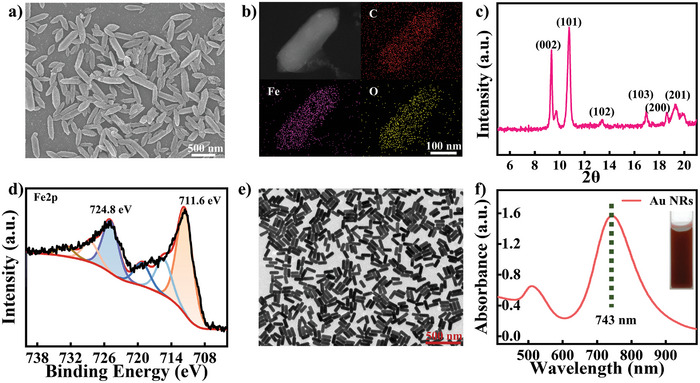
a,b) SEM and element mapping of MIL‐88B(Fe), c,d) XRD and XPS spectra of MIL‐88B(Fe), e,f) TEM and absorbance spectra of Au NRs (inset: the color of Au NRs solution).

The Fe‐MOF materials (MIL‐88B) exhibited a well‐defined rod‐like crystallographic structure, with a width of approximately 100 nm and a length ranging from 150 nm to 1 µm (**Figure** **1**a). The crystal structure illustrated in Figure S1a,b (Supporting Information) unveils the intricate arrangement of atoms within MIL‐88B(Fe), showcasing the precise positioning of iron ions and the organic ligands that seamlessly interconnect to forge a porous network. A uniform distribution of Fe, C, and O was observed within the material (Figure 1b). The FTIR spectrum revealed characteristic peaks at wavenumbers 1658, 1599, 1387, 747, and 549 cm^−1^ (Figure S1c, Supporting Information), corresponding to stretching vibrations of C═O bonds, asymmetric vibrations of C═O bonds, symmetric vibrations of C═O bonds, bending vibrations of C─H bonds in benzene molecules, and stretching vibrations of Fe─O bonds. The XRD signals demonstrated in Figure 1c are consistent with previous literature reports.^[^ ^24^, ^25^, ^26^ ^]^ The XPS analysis further confirms that the iron present in MIL‐88B(Fe) resides in a trivalent state, as depicted in Figure 1d. Examining the O1s spectrum (Figure S1e, Supporting Information), we observe three prominent characteristic peaks at 530.2, 531.8, and 533.3 eV, which can be distinctly attributed to the Fe─O, C═O, and O─H bonds, respectively.^[^ ^36^ ^]^ Similarly, analyzing the C1s spectrum (Figure S1f, Supporting Information) reveals three significant characteristic peaks at 284.6, 285.2, and 288.7 eV, which are ascribable to the C═C, C─C, and C═O bonds.^[^ ^37^ ^]^ Additionally, the synthesized Au NRs exhibited a homogeneous morphology, described as a rod‐like structure with a width of 35 nm and a length of about 105 nm (Figure 1e). Figure 1f shows the longitudinal localized surface plasmon resonance (LSPR) peak of Au NRs at 743 nm in a reddish brown solution (inset Figure 1f). It appears that both MIL‐88B(Fe) nanoenzymes and Au NRs have been successfully synthesized.

### 2.2 Feasibility of Building Multicolor Visualization Platform

**Figure 2:**
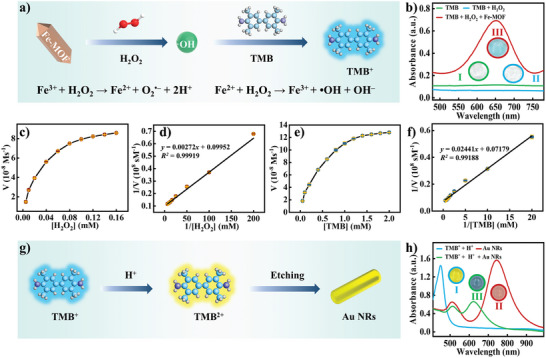
a) Diagram illustrating MIL‐88B(Fe) catalytic feasibility, b) absorbance spectra of TMB under different conditions, c,d) steady‐state kinetic assay and double reciprocal plots of MIL‐88B(Fe) at 0.5 × 10^−3^ m TMB and varying H_2_O_2_ concentrations, e,f) Steady‐state kinetic assay and double reciprocal plots of MIL‐88B(Fe) at 0.1 × 10^−3^ m H_2_O_2_ and different TMB concentrations, g) diagram showcasing Au NRs etching feasibility, h) absorbance spectra of TMB^+^ + H^+^, Au NRs and TMB^+^ + H^+^ + Au NRs.

The multicolor visualization platform involves two main processes: catalysis and etching. **Figure** **2**a provides a schematic illustration for evaluating the feasibility of catalysis. During the reaction process, H_2_O_2_ attaches itself to the iron site, facilitating an electron/charge transfer between H_2_O_2_ and MIL‐88B(Fe). This interaction leads to the reduction of Fe^3+^ on MIL‐88B(Fe) to Fe^2+^. Subsequently, the newly formed Fe^2+^ on the surface reacts with H_2_O_2_, generating Fe^3+^ and reactive oxygen species.^[^ ^38^ ^]^ As shown in Figure S2 (Supporting Information), after undergoing a Fenton‐like reaction, the BE value of Fe2p slightly decrease to 724.1 and 711.2 eV. Deconvolution of the Fe2p3/2 peak of after reaction MIL‐88B(Fe) reveals the presence of Fe^2+^ on the surface, suggesting that a portion of Fe^3+^ was reduced to Fe^2+^ during the reaction. Alternatively, as vividly illustrated in Figure S3 (Supporting Information), the structure of MIL‐88B(Fe) remains remarkably intact following the reaction. This underscores the material's exceptional cyclic stability, indicating that it retains both its structural integrity and catalytic properties. Furthermore, to ascertain which specific reactive oxygen species are produced by this reaction, we conducted both a free radical scavenging test and electron spin resonance (ESR) spectroscopy. As depicted in Figure S4a–c (Supporting Information), terephthalic acid (TPA), p‐benzoquinone (PBQ), and tryptophan (Try) served as scavengers for ·OH, superoxide anions (O_2_^∙−^), and singlet oxygen (^1^O_2_), respectively. By comparing these results, it becomes evident that the ·OH scavenger undergoes the most significant change post‐reaction, suggesting that ·OH is the primary reactive oxygen species generated. Furthermore, the ESR test results presented in Figure S4d–f (Supporting Information) confirm this conclusion, as the detection probe solely captured the signal of ·OH, providing additional verification and explanation. The absorption peak of TMB^+^ at 652 nm is not observed when it is added alone to H_2_O_2_ (Figure 2b). When MIL‐88B(Fe) was added to the solution, a peak of absorption at 652 nm was observed, and the solution color changed from colorless to blue. This indicates that MIL‐88B(Fe) with a peroxide‐like activity successfully converts TMB to TMB^+^. Further investigation was conducted on the steady‐state kinetics of MIL‐88B(Fe) enzyme activity in the presence of TMB and H_2_O_2_. TMB and H_2_O_2_ concentration are key factors that influence MIL‐88B(Fe) catalytic performance (Figure 2c–f). The enzyme exhibits a Michaelis‐Menten behavior similar to HRP, with a considerable increase in affinity (*K*_m_ value of 0.027 × 10^−3^ m) when H_2_O_2_ is used as the substrate (Table S1, Supporting Information). Additionally, MIL‐88B(Fe) demonstrates a relatively high affinity for TMB (*K*_m_ value of 0.340 × 10^−3^ m), indicating its efficient binding to the TMB mediator. It should also be noted that MIL‐88B(Fe) demonstrates a relatively high affinity for the substrate compared to other nanozymes, which may facilitate its tight binding to its active site during the reaction. The high affinity of MIL‐88B(Fe) for both TMB and H_2_O_2_ suggests its potential for use in biosensing applications.

Figure 2g illustrates that etching is feasible under acidic conditions, where TMB^+^ can be converted to the highly oxidative TMB^2+^, subsequently etching the Au NRs and affecting their aspect ratio. A change in absorption peak and color is observed following the conversion of TMB^+^ to TMB^2+^ upon HCl addition (Figure 2h‐I). Figure 2h‐II depicts the original absorption peak of Au NRs, which exhibits a significant color change after being etched by TMB^2+^ (Figure 2h‐III), transitioning from orange‐red to blue. The observed change in absorption peak and color is observed in Figure 2h provides compelling evidence for the successful etching of Au NRs using the colorimetric method. In addition to confirming the conversion of TMB^+^ to TMB^2+^ and the subsequent etching of the Au NRs, this change offers a visual indication of the etching process, making it easy to study color changes during biosensor etching reactions.

### 2.3 Feasibility Analysis of Sar by Intelligent Multicolor Visualization Platform

**Figure 3:**
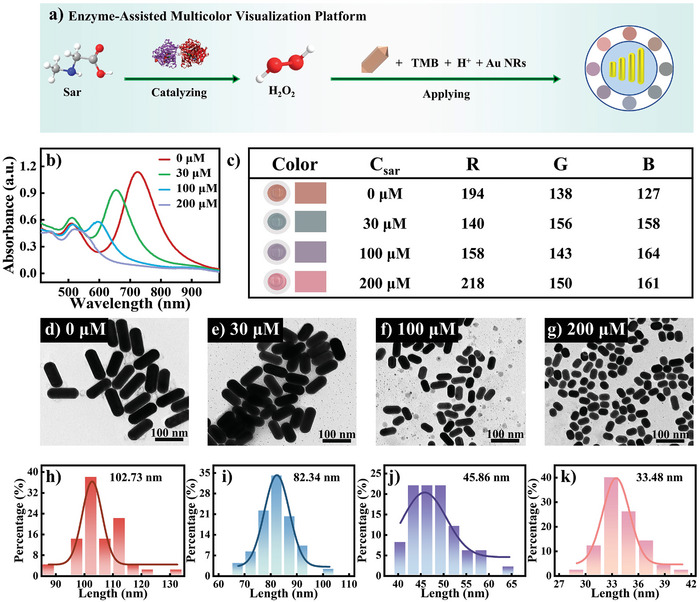
a) Diagram illustrating the colorimetric analysis of Sar, b) absorbance spectra of Au NRs at different concentrations of Sar, c) color photos and RGB values of various Sar concentrations, d–g) TEM images of Au NRs after reacting with different concentrations of Sar, h–g) corresponding particle size statistics.

The multicolor visualization platform is developed after Sar is catalyzed by SOX and hydrolyzed to generate H_2_O_2_ (**Figure** **3**a). An evaluation of its feasibility was performed using varying concentrations of Sar as a substitute for H_2_O_2_ under SOX catalysis. An increase in Sar concentration resulted in a gradual enhancement of the blue shift of Au NRs' longitudinal LSPR peak (Figure 3b). The transition from orange‐red to blue, purple, and pink was accompanied by diverse and pronounced color changes (Figure 3c). Leveraging smartphone technology, we extracted RGB values from these color changes and quantified them in digital form. The TEM images intuitively demonstrate the size variations in Au NRs following etching (Figure 3d–g). Increased Sar concentration results in elevated TMB^2+^ levels and more extensive etching of Au NRs, resulting in their gradual loss of length (Figure 3h–k). The changing shape and size of Au NRs, transitioning from rod‐shaped to shorter rods and ultimately to spherical shapes, serves as evidence that they can be sensitively detected through their longitudinal LSPR absorption peak. The color changes observed in the multicolor visualization platform provide a reliable and intuitive method for detecting Au NRs, corresponding to variations in size and shape. Through the quantitative analysis of the RGB values extracted from these color changes with smartphone technology, the presence and concentration of Sar can be accurately determined, making this platform an invaluable tool for sensitive detection.

### 2.4 Condition Optimization of Intelligent Multicolor Visualization Platform

Optimal experimental conditions were achieved by optimizing hexadecyl trimethyl ammonium bromide (CTAB) concentration, HCl concentration, MIL‐88B(Fe) concentration, and etching time. This study evaluated and compared the longitudinal LSPR absorption peak and the color change of the Au NRs system when subjected to 150 × 10^−6^ m Sar. The results indicated that the selected optimal conditions resulted in a purple color with a noticeable shift (Δ*λ*) in the longitudinal LSPR peak, while maintaining a distinct peak shape. Considering that CTAB is the primary factor influencing Au NR etching by TMB^2+^, we adjusted the CTAB concentration as shown in Figure S5a (Supporting Information). This increase gradually diminished as CTAB concentration rose, enabling us to select 75 mM as the optimal reaction concentration before observing minimal further increases. Optimizing the concentrations of HCl and MIL‐88B(Fe), as well as the etching time, was paramount to achieving the desired etching effect in our experiments. The concentration of MIL‐88B(Fe) has a direct influence on the generation of ·OH radicals by H_2_O_2_, which subsequently impacts the production of TMB^+^. And the HCl concentration plays a pivotal role in facilitating the transformation of TMB^+^ into TMB^2+^, a critical step within our reaction pathway. Lastly, the reaction time stands as a crucial determinant of the sample's ultimate etching effect. To pinpoint the optimal conditions for our experiment, we conducted a series of systematic optimizations, adhering to principles akin to those used for optimizing TMB. Through meticulous experimentation and analysis, we discerned that a HCl concentration of 200 × 10^−3^ m (Figure S5b, Supporting Information), a MIL‐88B(Fe) concentration of 20 µg mL^−1^ (Figure S5c, Supporting Information), and an etching time of 5 min (Figure S5d, Supporting Information) yielded the most favorable outcomes. Given the better catalytic activity of MIL‐88B(Fe) under acidic conditions and its susceptibility to damage under alkaline conditions, we selected a buffer with a pH value of 4.5 for constructing our catalytic system. This optimized system allowed us to fabricate an efficient and reproducible multicolor visualization platform that exhibits improved detection performance.

### 2.5 Quantitative Analysis of Sar by Intelligent Multicolor Visualization Platform

**Figure 4:**
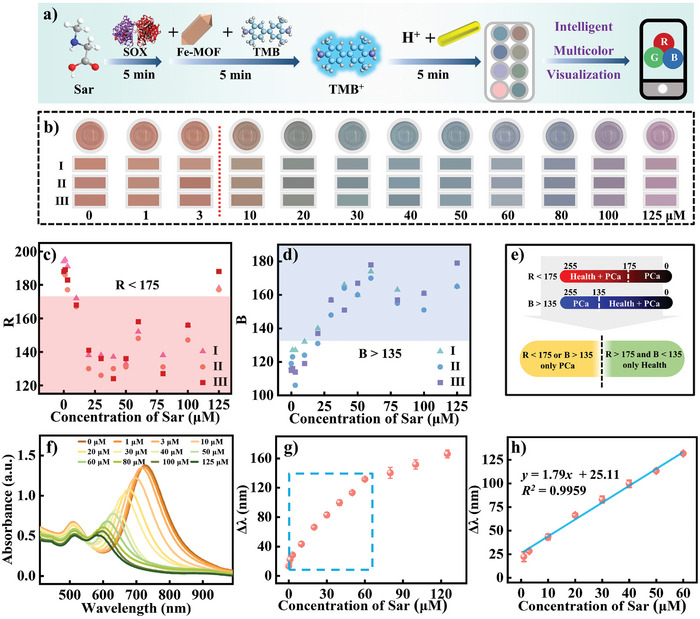
a) Diagram illustrating the colorimetric analysis of Sar, b) Photographs showcasing the Sar concentration‐dependent color and RGB values of Au NRs, c) diagnosing PCa based on *R* value, d) diagnosing PCa based on *B* value, e) diagnosing PCa based on both *R* and *B* value, f,g) absorbance spectra and respective Δ*λ* values of Au NRs at various concentrations of Sar, h) a linear relationship between Δ*λ* and Sar concentrations ranging from 1 × 10^−6^ m to 60 × 10^−6^ m (*R^2^* = 0.9959).

As illustrated in **Figure** **4**a, the reaction system was utilized to detect various concentrations of the biomarker Sar under optimal experimental conditions. With increasing concentrations of Sar (Figure 4b), the Au NRs solution exhibited a rich array color changes, transitioning from orange‐red to brown, gray, dark green, cyan‐blue, blue, purple, and finally pink‐purple. However, naked‐eye identification can be subjective and prone to misjudgment. Therefore, we attempted to extract RGB values from the solution using a smartphone (Table S2, Supporting Information). It should be noted that smartphone colors displayed slight variations due to factors such as external light interference and container construction, resulting in fluctuations in RGB values within a particular range. To minimize operational errors within this range, three random points were selected for color extraction.

Under healthy physiological condition, an order of magnitude or more increase in Sar levels in urine is typical (1–3 × 10^−6^ m), but these levels can be significantly enhanced under pathological circumstances such as PCa. Based on this information, we defined a range of 0–3 × 10^−6^ m Sar as a normal concentration level, with anything exceeding that level considered abnormal. At normal concentration levels, the *R*‐values in Au NRs solutions tended to be higher than 175, while the B‐values tended to be lower than 135. The *R*‐value fluctuations became larger with increasing Sar concentrations, exhibiting a general trend towards decline followed by an increase (Figure 4c). In contrast, *B*‐value fluctuations were relatively small, showing an overall trend towards continual growth (Figure 4d). The *G*‐value will not be analyzed due to the absence of an obvious change pattern (Figure S6, Supporting Information). The results of this study indicated that *R*‐values at high Sar concentrations would be close to those at low Sar concentrations, while *B*‐values at normal Sar concentrations would be similar to those at high Sar concentrations. Accordingly, in the case of *R* > 175 and *B* < 135, it is considered that Sar is within normal range of concentrations, while *R* < 175 or *B* > 135 could indicate a preliminary diagnosis of PCa. Overall, these results provide an intelligent useful tool for doctors to assess Sar concentrations and screening PCa.

In addition to changing colors, the longitudinal LSPR absorption peak of Au NRs in absorbance spectra showed variations (Figure 4f), with its shift increasing as Sar concentration increased. A linear relationship was observed between the LSPR peak shift caused by etching of Au NRs and Sar concentration within the concentration range of 1–60 × 10^−6^ m. The linear equation was *y* = 25.11 + 1.79*x* (*R* ^2^ > 0.9959) where *x* represents Sar concentration and y represents the Δλ. Using the formula LOD = 3*σ*/*S*, we have calculated a detection limit of 0.12 × 10^−6^ m, ensuring our method is highly sensitive and precise. The linear relationship between the LSPR peak shift and Sar concentration indicates that the change in absorbance spectra of Au NRs can be used as a reliable and quantitative measure of Sar concentration. This finding is significant as it suggests the potential for the development of a precise and accurate method for detecting and analyzing Sar levels, which could be crucial for early detection of PCa.

### 2.6 Intelligent Multicolor Visualization Platform of Selectivity and Anti‐Interference Properties

To assess the selectivity of our multicolor visualization platform, we substituted 60 × 10^−6^ m Sar with eight commonly occurring amino acids found in urine, each at equimolar concentrations: glycine, lysine, histidine, glutamate, arginine, tyrosine, cysteine, and tryptophan. As illustrated in Figure S7a (Supporting Information), the results demonstrated minimal shifts in the longitudinal LSPR peak of Au NRs when these eight amino acids replaced Sar. This underscores the robust specificity of the SOX‐catalyzed hydrolysis of Sar and highlights the exceptional selectivity of our multicolor visualization platform. To further evaluate the interference resistance of our colorimetric system, we introduced seven common ions typically present in urine environments (Na^+^, Mg^2+^, K^+^, NH_4_^+^, SO_4_^2−^, HCO_3_^−^, Cl^−^) along with three prevalent molecules (glucose, uric acid, urea) into the system, each at a concentration of 10 × 10^−3^ m. The findings indicated minimal impact on the longitudinal LSPR peak of Au NRs by these ions and molecules, as depicted in Figure S7b (Supporting Information), highlighting strong interference resistance exhibited by this multicolor visualization platform. Moreover, we measured the adaptability of the sensors constructed above in real biological samples. The human urine was applied as the medium to perform the recovery tests. The obtained recovery rates were in the range of 95.52–105.84% with acceptable RSDs (Table S3, Supporting Information). These results confirmed the excellent applicability of the sensor in human urine samples. And the R and B values of its display color are also in line with the previously summarized rules (Figure S8, Supporting Information).

### 2.7 Sar Analysis in PCa Urine Samples by Intelligent Multicolor Visualization Platform

**Figure 5:**
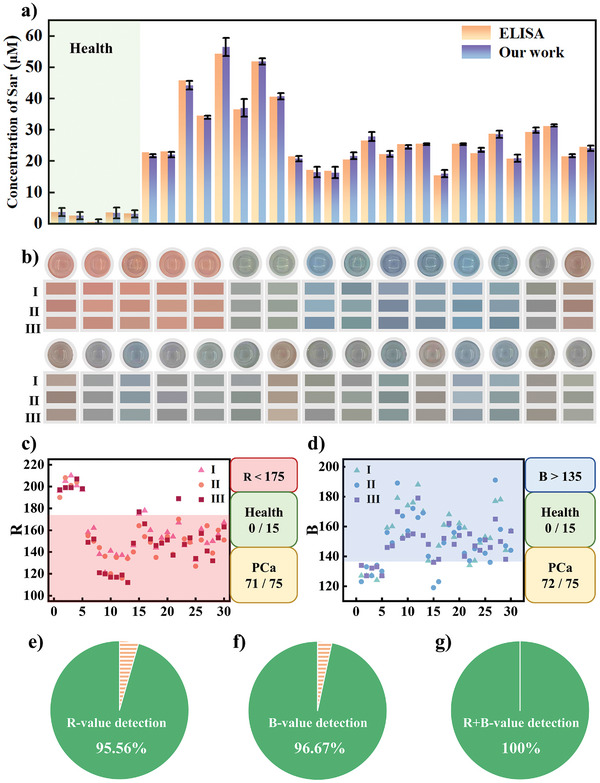
a) Comparison between urine sample test results and ELISA detection results, b) color photos and RGB values of different urine samples, c) diagnosing PCa based on *R* value, d) diagnosing PCa based on *B* value, e–g) accuracy of different RGB value detection methods.

Detection of Sar content in urine is important for PCa diagnosis and treatment, largely due to its noninvasive nature and potential for early detection. To assess the feasibility of this colorimetric method in real samples, we analyzed urine samples from five healthy individuals and 25 PCa patients. The results of these samples were also analyzed using commercially available Sar detection kits, and a comparison of the results shown in **Figure** **5**a. To further bolster the credibility of our detection method in comparison to kit detection, we have introduced the relative standard deviation (RSD) of Sar detection concentration as an additional performance metric. As illustrated in Table S4 (Supporting Information), the RSD value of our detection method mostly stays below 5%, unequivocally showcasing its remarkable accuracy and reliability. It is evident that colorimetric detection is comparable to commercially available test kits in terms of accuracy. The colorimetric results presented in Figure 5b indicate that urine samples from healthy individuals are orange‐red in color, while those from PCa patients are brown, gray, dark green, or blue. To minimize operational errors, the RGB analysis of each sample was carried out on three randomly selected color points. Based on the previously summarized patterns, if *R* > 175 indicates a healthy patient and *R* < 175 suggests PCa diagnosis, there is a accuracy rate of 96% in these diagnostic results (Figure 5c,e). Similarly, if *B* < 135 represents the presence of healthy patients and *B* > 135 represents the presence of PCa patients, the accuracy rate for these diagnostic results stands at 97% (Figure 5d,f). When both judgment criteria combined, the accuracy rate can reach as high as 100% within this limited sample experiment (Figure 5g). Additionally, the entire detection process, including analyzing the solution color, only takes 15 min. As Table S6 (Supporting Information) clearly demonstrates, our detection method stands out when compared to other recent sarcosine detection techniques, offering a superior detection limit and a more streamlined reaction time. These results demonstrate the potential of our intelligent kit to accurately and quickly diagnose PCa, and its accuracy could further improve with a larger sample size.

## 3 Conclusions

During this study, we developed a multicolor visualization intelligent kit for diagnosing PCa by detecting Sar in urine samples. Through the combined action of Fe‐MOFs and peroxidase, the simultaneous activity of SOX and Au NRs is controlled to produce a multicolored signal. A smartphone camera captures RGB color values, enabling direct and rapid diagnosis of PCa based on two specific RGB thresholds: *R* < 175 or *B* > 135. Furthermore, the multi‐color visualization bioassay kit demonstrated precise and rapid diagnostic capabilities in numerous patient urine samples, achieving a 100% accuracy rate for results. The specific RGB thresholds of *R* < 175 or *B* > 135 are crucial for PCa diagnosis as they serve as indicators of the presence or absence of specific biomarkers related to PCa. By setting these thresholds, the multicolor visualization intelligent kit can accurately identify and differentiate urine samples that contain elevated levels of these biomarkers, enabling a direct and rapid diagnosis of PCa. This approach eliminates the need for complex laboratory procedures and provides a reliable tool for early detection and monitoring of PCa in clinical settings.

## 4 Experimental Section

### Materials

The sarcosine (Sar, 99%) was purchased from Huzhen Industrial Co., Ltd. (Shanghai, China). Chloroauric acid (HAuCl_4_⋅3H_2_O, 99%) was obtained from Sigma Aldrich (Beijing, China). Hexadecyl trimethyl ammonium bromide (CTAB, 98.0%) was purchased from Aladdin Chemistry (Shanghai, China). 3,3′,5, 5′‐Tetramethylbenzidine (TMB, 98%) was purchased from Bidephar (Shanghai, China). Sarcosine oxidase (SOX) was acquired from Yuanye Bio‐Technology Co., Ltd. (Shanghai, China). 1,4‐Dicarboxybenzene (99%) was purchased from Innochem (Beijing, China). Ferric chloride hexahydrate (99.1%) was purchased from Leyan (Shanghai, China). Sodium borohydride (NaBH_4_, 99.0%), silver nitrate (AgNO_3_, >99.0%), ascorbic acid (AA, 99.7%), hydrochloric acid (HCl, 37 wt% in water), sodium hydroxide (NaOH, >96.0%), and N,N‐dimethylformamide (DMF, >99.0%), were purchased from Sinopharm Chemical Reagent Co., Ltd. (Shanghai, China). This experiment was conducted with ultrapure water obtained via the Colton system (resistivity greater than 18.2 MΩ cm^−1^). There is no need for further purification of any reagents or solvents used.

### Characterizations

The precision acidity meter (Shanghai Dapu Instrument Co., Ltd.), UV spectrophotometer (TU‐1810), S‐4800 scanning electron microscope (Hitachi, Japan), HT‐7700 transmission electron microscope (Hitachi, Japan), D8 Advance X‐ray diffractometer (Bruker, Germany), P60 Smartphone (Huawei, China) are utilized in the study.

### Synthesis of Au NRs

The Au NRs were synthesized using a seed‐mediated method.^[^ ^39^ ^]^ 1) Preparation of the seed solution: 0.25 mL of a 0.01 m HAuCl_4_ solution and 0.60 mL of a 0.01 m NaBH_4_ solution, prepared with ice‐cold water, were added to a 10 mL CTAB solution with a concentration of 0.10 m, followed by vigorous stirring at 1200 rpm for 2 min. Subsequently, the reaction mixture was allowed to stand at a temperature of 30 °C in a water bath for 2 h. 2) Growth of Au NRs: Under the conditions of a rotational speed of 600 rpm and a temperature of 30 °C in a water bath, the following reagents were sequentially added into a 120 mL CTAB solution (0.1 m): 1.2 mL of AgNO_3_ (0.01 m), 6 mL HAuCl_4_ (0.01 m), 0.96 mL ascorbic acid (vitamin C) solution (0.1 m), 2.4 mL HCl Solution (1 m), and finally, 96 µL gold seed solution. The reaction mixture was allowed to stand in the water bath at 30 °C for more than 6 h. Subsequently, the prepared Au NRs were centrifuged twice at 7000 rpm for 10 min each time to remove CTAB. Afterward, the precipitate was dispersed and concentrated into a solution with a volume of 45 mL using water.

### Synthesis of the Fe‐MOF

Ferric chloride hexahydrate (1.892 g) and 1,4‐benzenedicarboxylic acid (1.163 g) were subjected to hydrothermal treatment in DMF (50 mL) with NaOH (2 m, 4 mL) at 100 °C for 12 h. Following solvothermal treatment, the Fe‐MOF synthesized was washed with DMF and methanol for three times.

### Feasibility of Building Multicolor Visualization Platform

Mix 20 µL 1 × 10^−3^ m H_2_O_2_, 20 µL 10 × 10^−3^ m TMB, 10 µL 1 mg mL^−1^ MIL‐88B(Fe), and 150 µL HAc/NaAc buffer (pH = 4.5), and let the reaction stand for 10 min. Add 10 µL 6 m HCl and 50 µL Au NRs successively. Observe the change of color and absorption spectrum.

### Multicolor Visualization Platform for the Detection of Sar

First, 20 µL Sar (different concentrations) were mixed with 10 µL SOX (36 µg mL^−1^) and incubated in a water bath at 37 °C for 5 min. Next, add 75 µL of HAc/NaAc buffer (pH = 4.5), followed by the addition of 5 µL Fe‐MOF (20 µg mL^−1^) and 10 µL TMB (0.36 × 10^−3^ m). Incubate the mixture again in a water bath at 50 °C for another 5 min. Finally, sequentially add 10 µL of 200 × 10^−3^ m HCl, 100 µL 75 × 10^−3^ m CTAB, and 50 µL Au NRs solution, followed by etching in a water bath at 50 °C for 5 min. All concentrations mentioned in this experiment refer to the final concentrations. Subsequently, measure the absorption spectrum through spectrophotometer and capture a photograph of the resulting solution color by smartphone. Then RGB values of the color photos were extracted by the software (Adobe Photoshop CS6).

### Detection of Urine Samples from PCa Patients

Replace the Sar with the concentrated urine sample, which has undergone a 14‐fold increase in concentration. The remaining steps remain consistent with the aforementioned experimental procedures. This study strictly adhered to the ethical guidelines set by the Ethics Review Committee of [the First Affiliated Hospital of Ningbo University/China]. Prior to recruiting any participants, this study obtained approval from the Ethics Review Committee (Approval number of ethics: No. 109A, 2023 Research). All participants voluntarily signed the informed consent form after fully understanding the study's purpose, methods, potential risks, and benefits. This informed consent form detailed their rights, the anonymous processing of research data, and their freedom to withdraw from the study at any time. It is confirmed that informed written consent was obtained from all participants.

### Statistical Analysis

The recorded data were analyzed and processed utilizing Origin software, developed by OriginLab in Northampton, MA. The measurement data are presented as the mean ± standard deviation. The results are summarized as the mean standard deviation, based on three replicates (*n* = 3). Statistical significance was assigned to p‐values below 0.05. Unless specified otherwise in the text or legend, the characterization data, including spectrum and target signal values, for this study will not undergo further processing.

## Conflict of Interest

The authors declare no conflict of interest.

## Supporting information



Supporting Information

## Data Availability

Research data are not shared.
