# Supplementary material for: Intelligent Screening of Prostate Cancer Individuals Using an Enzyme‐Assisted Multicolor Visualization Platform
Source: Adv Sci (Weinh). 2024 Nov 8;12(1):2408825. doi: 10.1002/advs.202408825 (PMC11714164; doi:10.1002/advs.202408825)
Supplement: Supplementary file 1 — Supporting Information [file ADVS-12-2408825-s001.docx]

**Supporting Information**

**Intelligent Screening of Prostate Cancer Individuals Using an** **Enzyme-Assisted Multicolor Visualization Platform**

Ruomei Teng^a#^, Ming Li^a,b#^, Zikang Chen^a^, Jianli Lin^a^, Yuhan Zhang^a^, Hang Li^a^, Zejun Yan^b^, Dingyuan Zhang^a^, Caiping Ding^a^*, and Youju Huang^a^*

^a^ College of Material, Chemistry and Chemical Engineering, Key Laboratory of Organosilicon Chemistry and Material Technology, Ministry of Education, Department of Orthopedics, Hangzhou Normal University affiliated Hospital, Hangzhou Normal University, Hangzhou, 311121, Zhejiang, China.

^b^ Department of Urology & Nephrology, The First Affiliated Hospital of Ningbo University, 59, Liuting Street, Ningbo, 315010, Zhejiang, China

^#^Ruomei Teng and Ming Li contributed equally to this work.

*Corresponding author:

Caiping Ding, E-mail: cpding@hznu.edu.cn

Youju Huang, E-mail: yjhuang@hznu.edu.cn


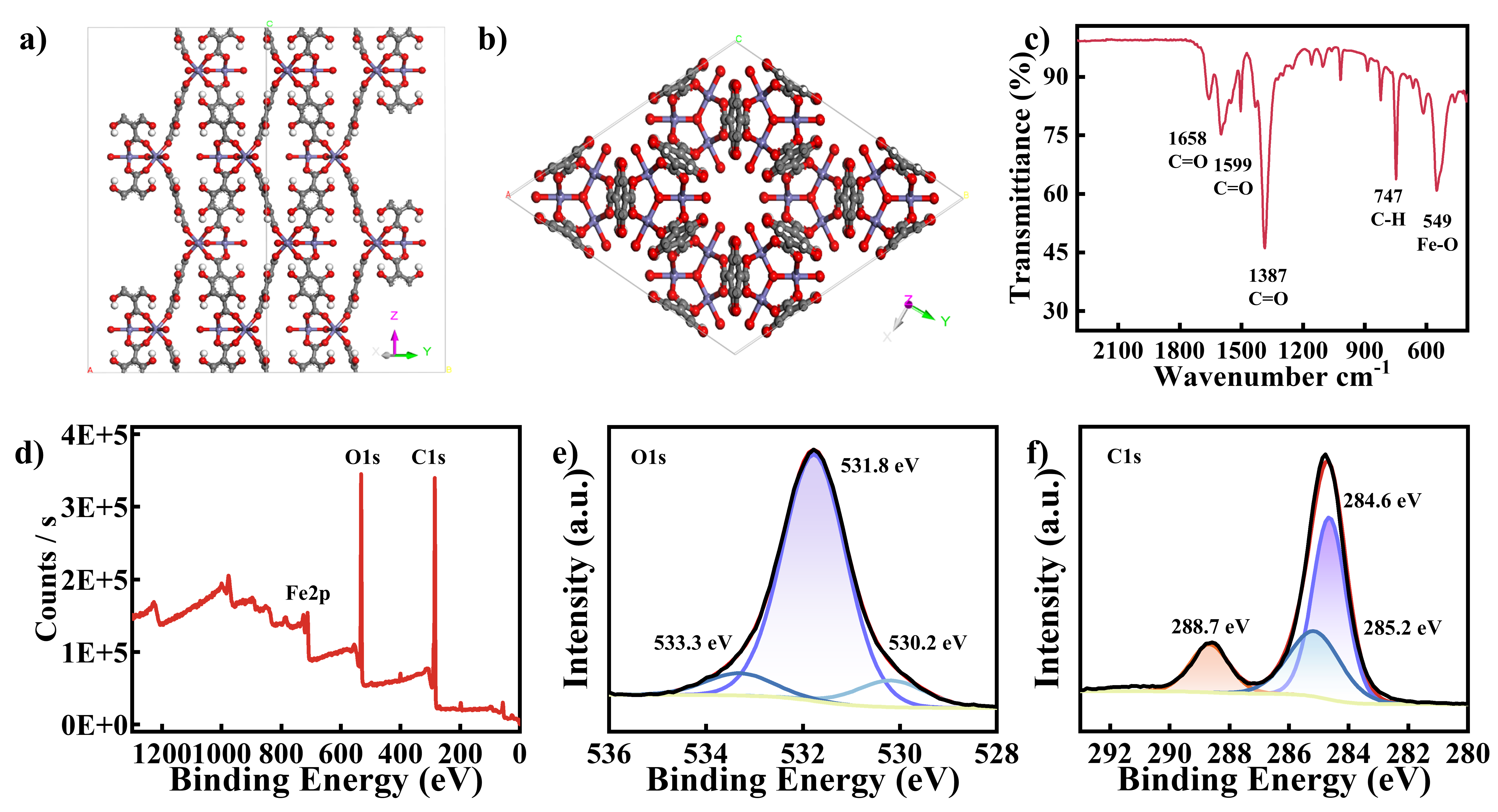


**Figure S1.** Representation of MIL-88B(Fe). (a, b) Crystalline structure, (c) FTIR spectrum, (d-e) XPS spectra: (d) survey spectrum, (e) C1s, (f) O1s.


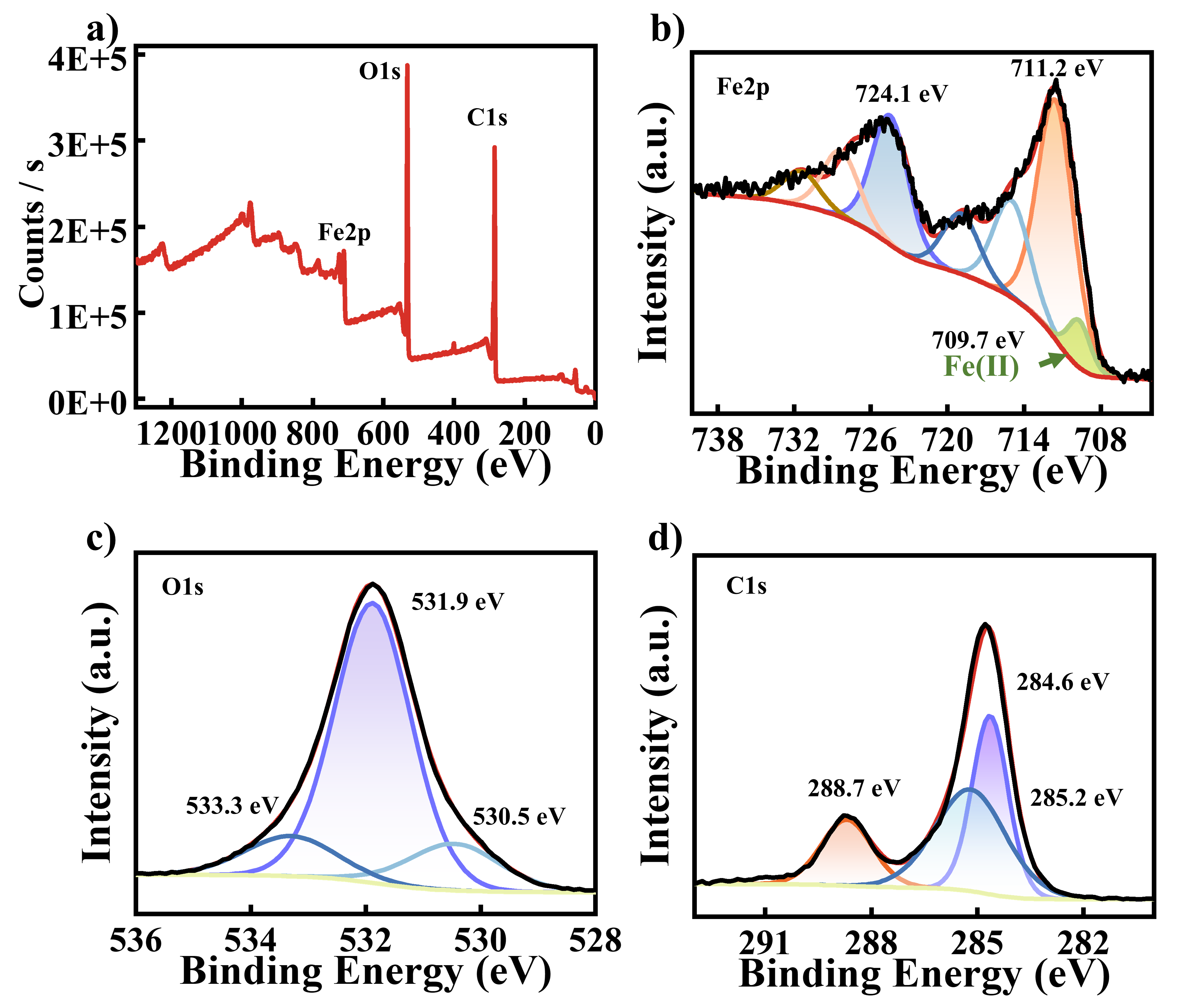


**Figure S2.** XPS spectra of MIL-88B(Fe) after reaction. (a) survey spectrum, (b) Fe2p, (c) O1s, (d) C1s.


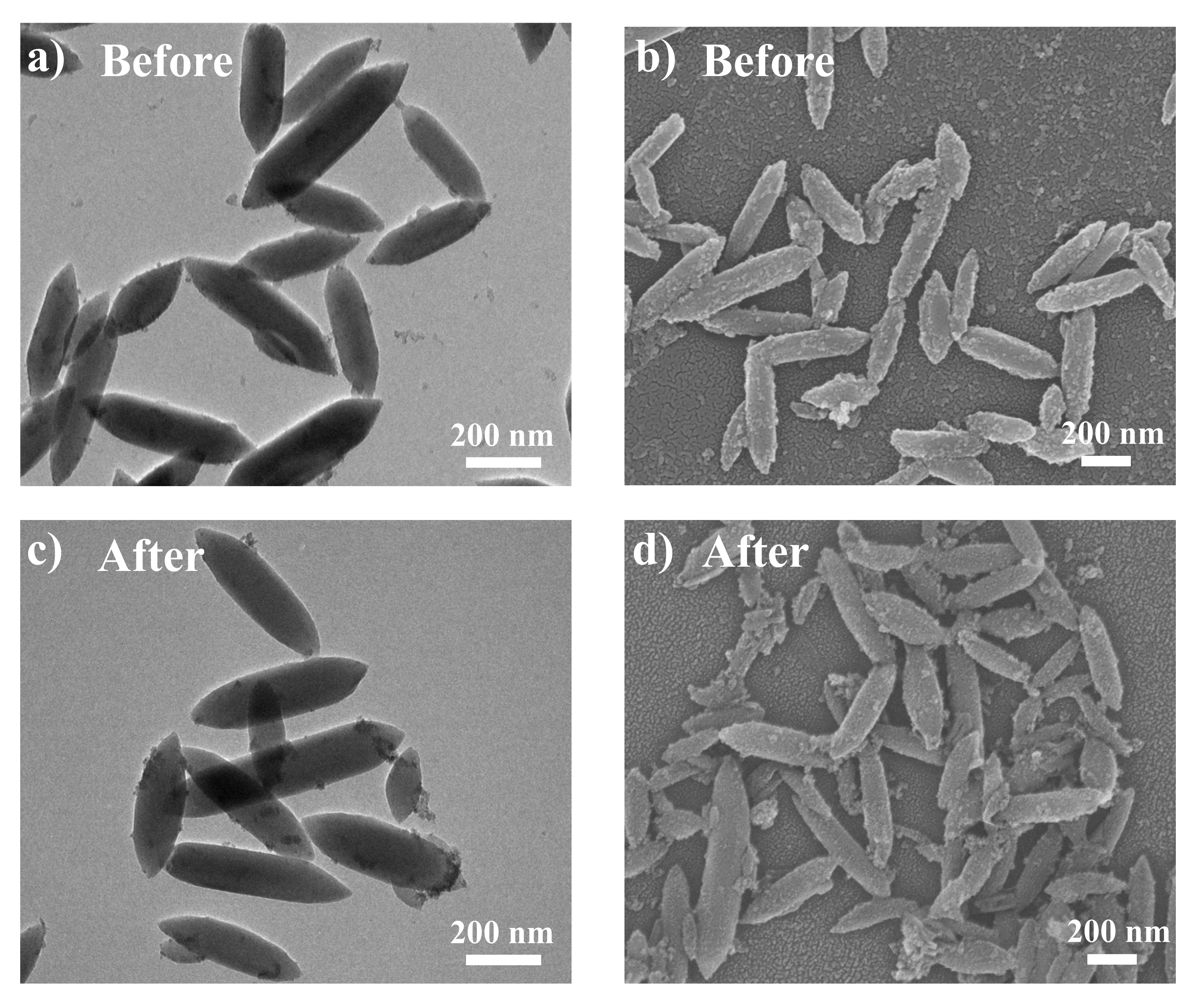


**Figure S3.** Structures of MIL-88B(Fe) both before and after undergoing a catalytic reaction. (a, b) TEM and SEM of MIL-88B(Fe) before reaction, (c, d) TEM and SEM of MIL-88B(Fe) after reaction.


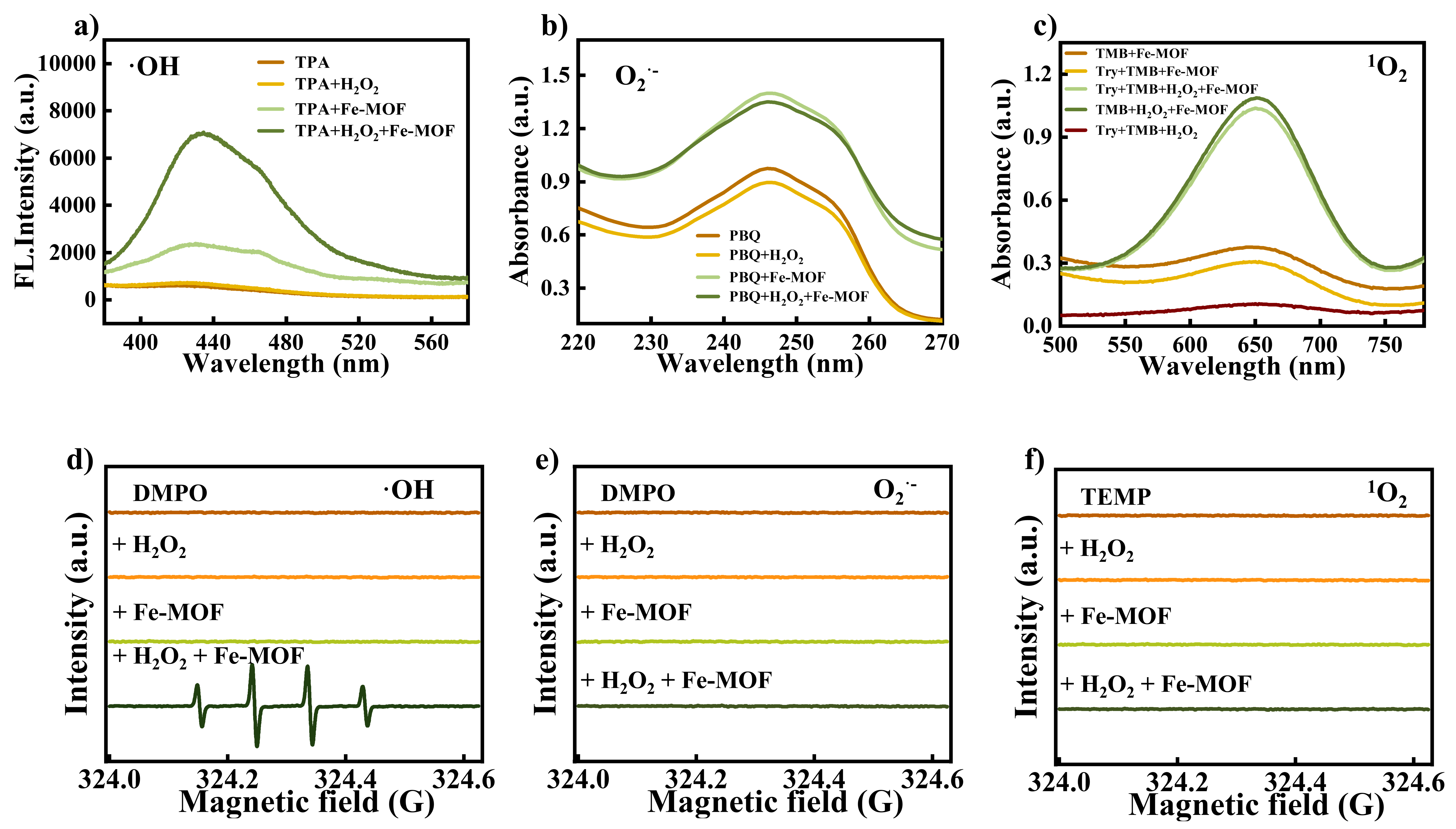


**Figure S4.** Verification of ROS. (a) Fluorescence spectra of TPA to verify **·**OH, (b) Absorbance spectra of PBQ to verify O_2_**^·^**^-^, (c) Absorbance spectra of TMB to verify ^1^O_2_, (d) ESR spectra to verify ·OH, (e) ESR spectra to verify O_2_**^·^**^-^, (f) ESR spectra to verify ^1^O_2_.

**Table S1.** Comparison of Michaelis Constant (*K*_m_).

|  | H_2_O_2_  *K*_m_ (mM） | TMB  *K*_m_ (mM) | Ref. |
| --- | --- | --- | --- |
| HRP | 3.7 | 0.434 | [1] |
| Fe_3_O_4_ MNPs | 154 | 0.098 | [1] |
| 10 Fe_2_O_3_ CNTs | 0.704 | 0.515 | [2] |
| Mesoporous Fe_2_O_3_ | 146.7 | 0.298 | [3] |
| Cu-MOF | 8.46 | 0.120 | [4] |
| Zr-MOF | 0.59 | 0.05 | [5] |
| MIL-101 (Fe) | 0.043 | 0.585 | [6] |
| MIL-88B (Fe) | 0.027 | 0.340 | This work |


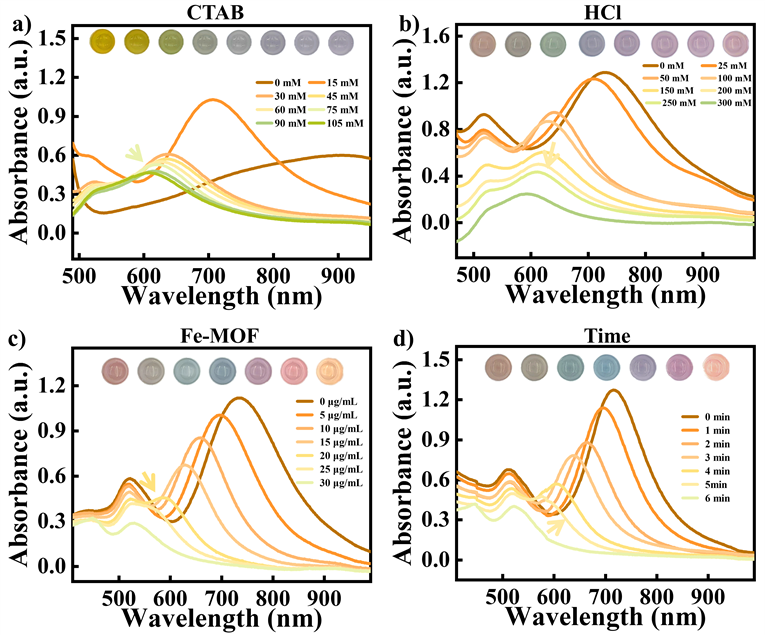


**Figure S5.** Absorbance spectra to optimize the reaction conditions. (a) CTAB concentration, (b) HCl concentration, (c) Fe-MOF concentration, (d) Etching time. (The illustration is the corresponding color).

**Table S2.** The RGB value of Figure 4b.

| C_sar_ μM | R-I | G-I | B-I | R-II | G-II | B-II | R-III | G-III | B-III |
| --- | --- | --- | --- | --- | --- | --- | --- | --- | --- |
| 0 | 194 | 135 | 117 | 187 | 131 | 119 | 188 | 127 | 115 |
| 1 | 195 | 135 | 127 | 186 | 138 | 123 | 189 | 133 | 116 |
| 3 | 191 | 145 | 127 | 177 | 122 | 106 | 183 | 127 | 114 |
| 10 | 172 | 150 | 132 | 167 | 141 | 124 | 168 | 136 | 119 |
| 20 | 138 | 143 | 140 | 130 | 132 | 131 | 141 | 143 | 137 |
| 30 | 138 | 152 | 157 | 126 | 143 | 148 | 136 | 156 | 157 |
| 40 | 137 | 156 | 166 | 130 | 152 | 164 | 124 | 142 | 151 |
| 50 | 133 | 152 | 160 | 131 | 148 | 160 | 136 | 156 | 167 |
| 60 | 152 | 164 | 174 | 148 | 156 | 170 | 158 | 168 | 178 |
| 80 | 138 | 144 | 163 | 131 | 138 | 155 | 127 | 137 | 157 |
| 100 | 156 | 142 | 161 | 147 | 131 | 151 | 156 | 141 | 161 |
| 125 | 178 | 148 | 165 | 177 | 142 | 165 | 188 | 157 | 179 |


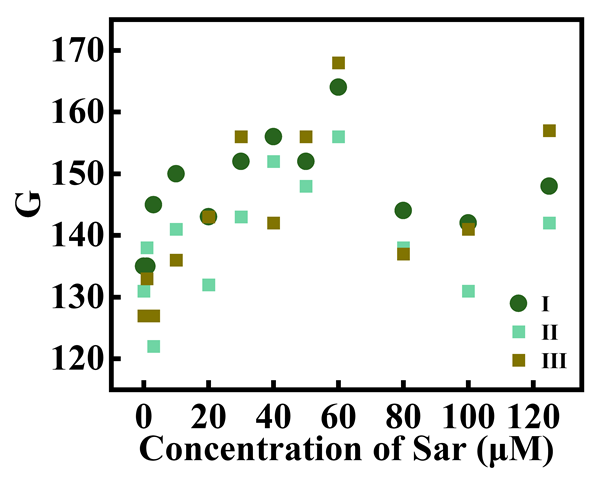


**Figure S6**. Sar concentration-dependent G values of Au NRs.


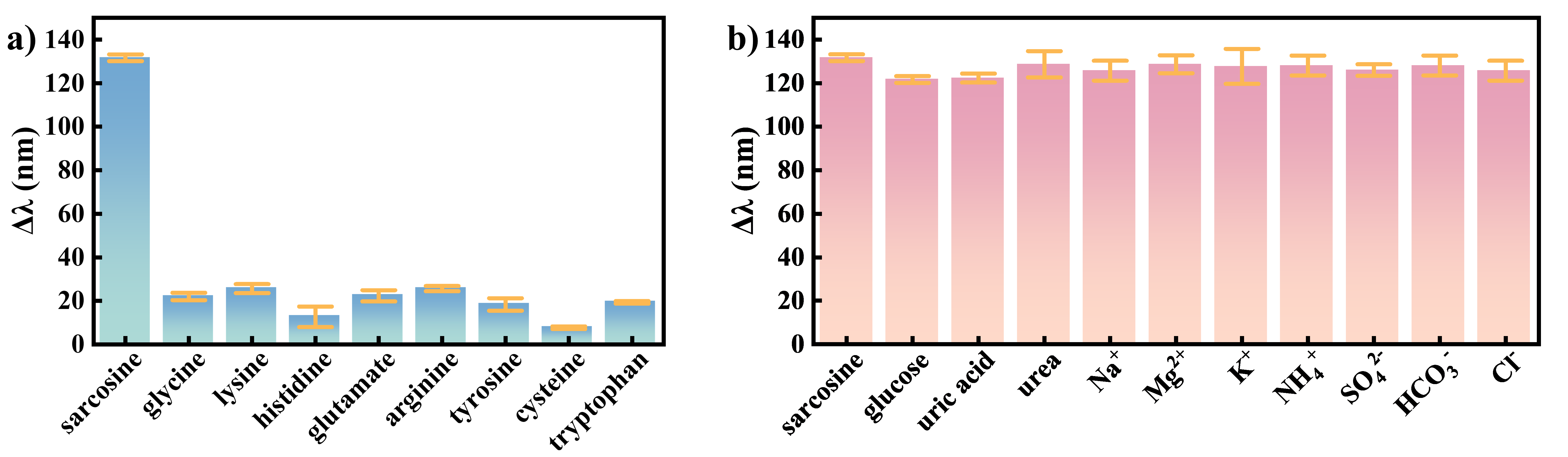


**Figure S7.** Respective Δλ values of Au NRs at different conditions to optimize specificity and anti-interference of this colorimetric sensor array. (a) Sar and eight common amino acids, (b) Sar with seven common ions present and three prevalent molecules in urine.

**Table S3.** Spike and recovery of Sar assay.

| Number | Sar in urine (μM) | Colorimetric Biosensors (μM) | Color  change | Recovery  (%) | RSD  (%) |
| --- | --- | --- | --- | --- | --- |
| 1 | 10 | 9.72 ± 0.39 | 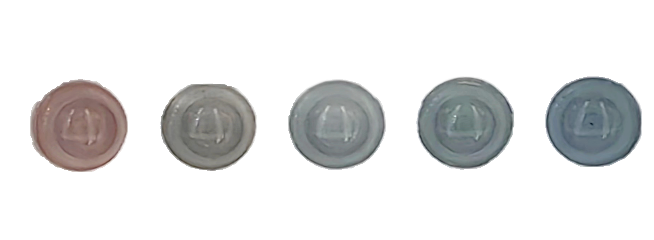 | 97.15 | 2.85 |
| 2 | 20 | 21.17 ± 0.79 |  | 105.84 | 5.84 |
| 3 | 30 | 31.22 ± 2.37 |  | 104.08 | 4.08 |
| 4 | 40 | 38.21 ± 1.97 |  | 95.52 | 4.48 |
| 5 | 50 | 48.82 ± 1.18 |  | 97.64 | 2.36 |


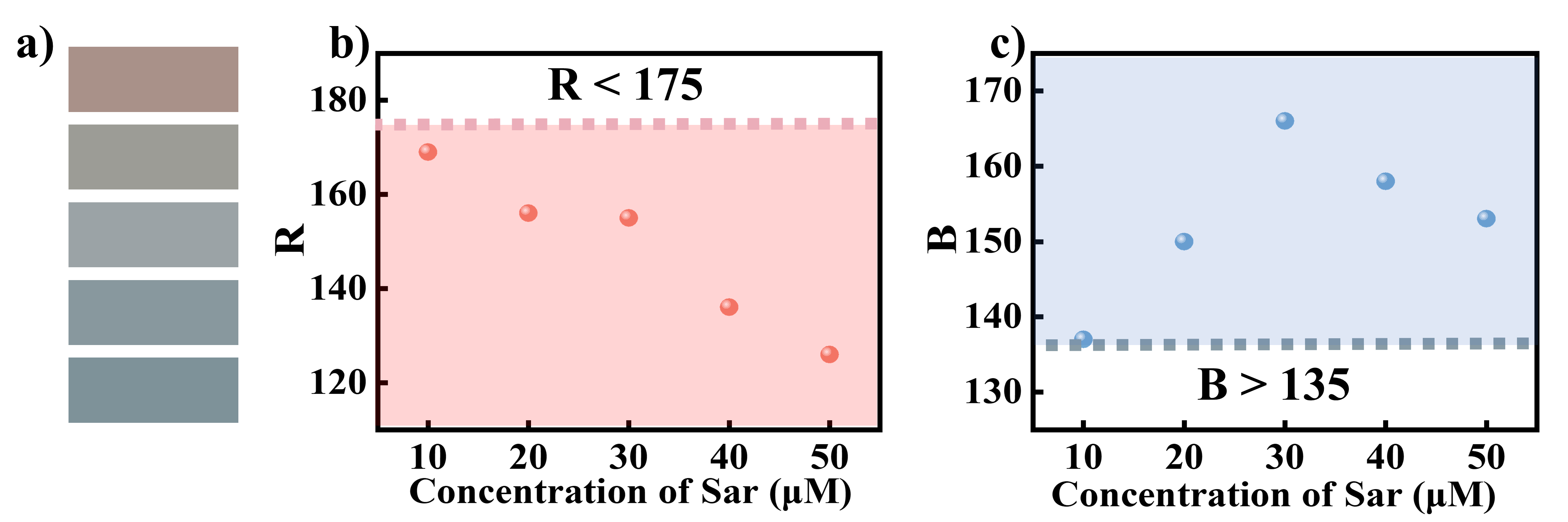


**Figure S8.** The Color analysis of spike and recovery experience. (a) The corresponding color, (b) R value, (c) B value.**Table S4.** RSD of Sar assay in Figure 5a.

| Sample  Number | ELISA  (μM) | This Work  (μM) | RSD  (%) |
| --- | --- | --- | --- |
| 1 | 3.72 | 3.66 ± 1.29 | 1.49 |
| 2 | 2.51 | 2.55 ± 1.16 | 1.38 |
| 3 | 0.50 | 0.50 ± 0.97 | 1.33 |
| 4 | 3.62 | 3.48 ± 1.71 | 4.04 |
| 5 | 3.25 | 3.10 ± 1.16 | 4.46 |
| 6 | 22.75 | 21.73 ± 0.56 | 4.51 |
| 7 | 23.07 | 22.10 ± 0.85 | 4.19 |
| 8 | 45.79 | 44.26 ± 1.41 | 3.33 |
| 9 | 34.55 | 34.02 ± 0.56 | 1.55 |
| 10 | 54.26 | 56.55 ± 2.87 | 4.21 |
| 11 | 36.62 | 37.00 ± 2.81 | 1.03 |
| 12 | 51.93 | 51.89 ± 0.97 | 0.07 |
| 13 | 40.62 | 40.72 ± 0.97 | 0.24 |
| 14 | 21.49 | 20.8 ± 0.85 | 3.25 |
| 15 | 17.18 | 16.51 ± 1.61 | 3.87 |
| 16 | 16.92 | 16.33 ± 1.80 | 3.54 |
| 17 | 20.53 | 21.73 ± 1.12 | 5.83 |
| 18 | 26.55 | 27.87 ± 1.48 | 4.97 |
| 19 | 22.21 | 22.28 ± 0.97 | 0.35 |
| 20 | 25.36 | 24.52 ± 0.56 | 3.32 |
| 21 | 25.52 | 25.45 ± 0.32 | 0.26 |
| 22 | 15.42 | 15.95 ± 1.16 | 3.45 |
| 23 | 25.51 | 25.45 ± 0.32 | 0.23 |
| 24 | 22.52 | 23.59 ± 0.65 | 4.75 |
| 25 | 28.81 | 28.62 ± 1.16 | 0.67 |
| 26 | 20.75 | 20.98 ± 1.16 | 1.13 |
| 27 | 29.38 | 29.92 ± 0.85 | 1.83 |
| 28 | 31.15 | 31.41 ± 0.32 | 0.84 |
| 29 | 21.53 | 21.73 ± 0.56 | 0.92 |
| 30 | 24.56 | 24.15 ± 0.85 | 1.69 |

**Table S5.** The R and B value of Figure 5b.

| Sample | R-I | B-I | R-II | B-II | R-III | B-III |
| --- | --- | --- | --- | --- | --- | --- |
| 1 | 196 | 127 | 190 | 123 | 197 | 134 |
| 2 | 205 | 127 | 208 | 133 | 199 | 127 |
| 3 | 210 | 127 | 201 | 127 | 199 | 132 |
| 4 | 201 | 124 | 204 | 134 | 207 | 133 |
| 5 | 197 | 127 | 198 | 130 | 198 | 127 |
| 6 | 158 | 159 | 155 | 156 | 149 | 146 |
| 7 | 162 | 160 | 150 | 149 | 152 | 147 |
| 8 | 138 | 179 | 144 | 189 | 121 | 170 |
| 9 | 121 | 153 | 136 | 167 | 120 | 152 |
| 10 | 141 | 174 | 120 | 157 | 117 | 154 |
| 11 | 137 | 176 | 135 | 172 | 117 | 155 |
| 12 | 137 | 188 | 116 | 166 | 119 | 179 |
| 13 | 135 | 170 | 134 | 169 | 112 | 154 |
| 14 | 146 | 137 | 140 | 140 | 148 | 148 |
| 15 | 175 | 136 | 164 | 119 | 177 | 136 |
| 16 | 178 | 147 | 154 | 123 | 166 | 138 |
| 17 | 160 | 161 | 149 | 149 | 152 | 152 |
| 18 | 144 | 168 | 135 | 161 | 146 | 162 |
| 19 | 153 | 153 | 149 | 156 | 148 | 148 |
| 20 | 158 | 162 | 154 | 159 | 151 | 154 |
| 21 | 154 | 159 | 138 | 142 | 137 | 140 |
| 22 | 167 | 134 | 170 | 137 | 189 | 155 |
| 23 | 146 | 137 | 152 | 145 | 147 | 147 |
| 24 | 149 | 149 | 149 | 151 | 153 | 144 |
| 25 | 133 | 153 | 127 | 142 | 134 | 151 |
| 26 | 161 | 152 | 151 | 136 | 157 | 146 |
| 27 | 148 | 177 | 164 | 191 | 141 | 165 |
| 28 | 150 | 178 | 139 | 158 | 132 | 150 |
| 29 | 156 | 144 | 160 | 147 | 153 | 138 |
| 30 | 167 | 157 | 151 | 144 | 163 | 157 |

**Table S6.** Comparison of other detection methods.

| Responsive material | Synthetic  material | | LOD | Detection  Time | Ref. |
| --- | --- | --- | --- | --- | --- |
| ABTS | CCA-YH | | 0.218 μM | 60 min | [7] |
| TMB | HRP | | 5 μM | 35 min | [8] |
| TMB | Fe_3_O_4_@SiO_2_@NiCo_2_S_4_ | | 0.42 μM | 60 min | [9] |
| TMB | SiO_2_@TiO_2_/ PDI-OH | | 0.12 μM | 32 min | [10] |
| TMB | HRP | | 1 μM | 30 min | [11] |
| TMB | CeO_2_ /Pt NPs | | 0.477 μM | 40 min | [12] |
| TCPP-Pt | TCPP-Pt/SOX@HMUiO | | 2.1 μM | 60 min | [13] |
| OPD | | MnO_2_NS | 0.36 μM | 104 min | [14] |
| Au NRs | | MIL-88B(Fe) | 0.12 μM | 15 min | This work |

**References**

[1] L. Gao, J. Zhuang, L. Nie, J. Zhang, Y. Zhang, N. Gu, T. Wang, J. Feng, D. Yang, S. Perrett, X. Yan, *Nat. Nanotechnol*. **2007**, *2*, 577.

[2] Y. Yang, T. Li, Y. Qin, L. Zhang, Y. Chen, *Front. Chem*. **2020**, *8*, 564968.

[3] R. Bhattacharjee, S. Tanaka, S. Moriam, M. K. Masud, J. Lin, S. M. Alshehri, T. Ahamad, R. R. Salunkhe, N. T. Nguyen, Y. Yamauchi, M. S. A. Hossain, M. J. A. Shiddiky, *J. Mater. Chem. B* **2018**, *6*, 4783.

[4] Q. Liu, Z. He, H. Wang, X. Feng, P. Han, *Microchim. Acta* **2020**, *187*, 524.

[5] L. Wang, Z. Hu, S. Wu, J. Pan, X. Xu, X. Niu, *Anal. Chim. Acta* **2020**, *1121*, 26.

[6] J. Guo, S. Wu, Y. Wang, M. Zhao, *Sensor. Actuat. B-chem*. **2020**, *312*, 128021.

[7] Z. Ma, L. Yang, Y. Wang, M. Wang, W. Qi, Z. *He, Chem. Eng. J.* **2021**, *416*, 129149.

[8] X. Yang, C. Jin, S. Yang, M. Tian, *Sensor. Actuat. B-Chem.* **2024**, *412*, 135849.

[9] X. Wang, M. Chen, L. Zhao, *Chem. Eng. J.* **2023**, *468,* 143612.

[10] Q. Liu, S. Cao, Q. Sun, C. Xing, W. Gao, X. Lu, X. Li, G. Yang, S. Yu, Y. Chen, *J. Hazard. Mater.* **2022**, 436, 129321.

[11] X. Yang, C. Jin, J. Zheng, F. Chai, M. Tian, *Sensor. Actuat. B-Chem.* **2023**, *394*, 134417.

[12] W. Li, T. Li, S. Chen, D. Deng, Y. Ji, R. Li, *Sensor. Actuat. B-Chem.* **2022**, *355*, 131341.

[13] L. Zhao, J. Yang, M. Gong, K. Li, J. Gu, *J. Am. Chem. Soc.* **2021**, *143*, 15145.

[14] Z. Yu, J. Tang, H. Gong, Y. Gao, Y. Zeng, D. Tang, X. Liu, *Adv. Funct. Mater.* **2023**, *33*, 2301457.
